# Clinical profile of reading ability and reading and writing achievement of children with borderline full-scale intellectual quotient: a prospective study

**DOI:** 10.1186/s12887-021-02865-z

**Published:** 2021-09-07

**Authors:** Riyo Ueda, Yoshimi Kaga, Yosuke Kita, Eiji Nakagawa, Takashi Okada, Masumi Inagaki

**Affiliations:** 1 Department of Developmental Disorders, National Institute of Mental Health, National Center of Neurology and Psychiatry, 4-1-1 Ogawahigashi-Cho, Kodaira, 187-8553 Tokyo, Japan; 2 Department of Child Neurology, National Center Hospital, National Center of Neurology and Psychiatry, 4-1-1 Ogawahigashi-Cho, Kodaira, 187-8551 Tokyo, Japan; 3 Department of Pediatrics, Faculty of Medicine, Yamanashi University, 1110, Shimokato, Chuo, Yamanashi Kofu, Japan; 4 Mori Arinori Center for Higher Education and Global Mobility, Hitotsubashi University, 2-1, Kunitachi, 186-8601 Tokyo, Japan; 5 Cognitive Brain Research Unit (CBRU), Faculty of Medicine, University of Helsinki, Haartmaninkatu 3, FI-00290 Helsinki, Finland

**Keywords:** Developmental dyslexia, Reading ability, Intellectual quotient, Academic achievement, Age

## Abstract

**Background:**

Poor reading ability is one of the common causes of low academic performance. In previous studies, children with dyslexia were found to demonstrate poor academic achievement due to poor reading ability. However, the relationship between academic achievement and reading ability in children with a borderline full-scale intellectual quotient (FSIQ) is unknown. This study aimed to clarify the clinical characteristics of children with borderline FSIQ and poor reading ability, and differentiate these characteristics from those of children with higher FSIQ and poor reading ability.

**Methods:**

A total of 126 children (aged 6–15 years) identified as having low academic performance were enrolled. The reading ability of children was assessed through their performance on *the hiragana* (Japanese syllabary) reading task, while their reading and writing achievement was assessed through their reading and writing score on the Kaufman Assessment Battery for Children, Second Edition. Children were categorized into two groups based on their FSIQ score (FSIQ > 85 and 85 ≥ FSIQ ≥ 70). Reading ability in children was evaluated by referring to the linear relationship between FSIQ and the standard deviation value of reading tasks in typically developing children. A one-way analysis of variance (ANOVA) was performed to examine clinical characteristics between higher and lower FSIQ groups. Associations between reading and writing achievement, reading ability, and ages of children were assessed using Pearson’s product-moment correlation coefficients for the higher and lower FSIQ groups.

**Results:**

Poorer reading and writing achievement was associated with poorer reading ability in the higher FSIQ group. Conversely, poorer reading and writing achievement and poor reading ability were associated with older age in the lower FSIQ group.

**Conclusions:**

Poor reading and writing achievement were associated with older age, not with poor reading ability in the lower FSIQ group. Children with lower FSIQ need appropriate educational interventions based on independent assessments to further their academic achievement and reading ability. Moreover, they need more frequent evaluations of their academic achievement than do children with higher FSIQ and poor reading ability since they are more likely to be at a lower academic achievement level at an older age.

**Supplementary Information:**

The online version contains supplementary material available at 10.1186/s12887-021-02865-z.

## Background

There are many causes for low academic performance in school-aged children [1–6]. For those with developmental dyslexia, a neurodevelopmental disorder, poor reading ability is one of the most common and severe causes for it [4–6].

Developmental dyslexia is the most common specific learning disorder, characterized by difficulties in learning to decode (read aloud) and spell. The prevalence of dyslexia among children at the end of their primary education has been determined to be approximately 4–5 % in both Dutch and American population-based studies [7]. A national longitudinal survey in Japan revealed that less than 2 % of children had reading delay/impairment by the time they reached the final grade of primary school. The prevalence of dyslexia in Japan was estimated to be lower than that in foreign countries in which alphabets were used [8]. According to the International Dyslexia Association, dyslexia is marked by impairment in accurate and/or fluent word recognition, and spelling and decoding abilities. Even though children with dyslexia have the same educational and sociocultural resources as typically developing children (TDC), their reading abilities are below the levels expected for their chronological ages [4]. In Japan, children with dyslexia are diagnosed by poor reading ability (prolonged reading time) of *hiragana* (Japanese syllabary), frequent overall poor reading and writing performance with mixed Chinese character and *hiragana* than TDC; therefore, acquirement of vocabulary and knowledge is restricted [9]. It has been speculated that one of the main causes of reading disabilities is impairment in phonological processing [10]. Children with dyslexia have been found to demonstrate poor academic achievement due to poor reading ability [4–6].

In addition, similar reading disabilities compared to those of children with dyslexia have been found in children with a low full-scale intellectual quotient (FSIQ) in previous studies [10–12]. Until recently, it was unclear if the reading ability of such children fell under the same spectrum as that of children with dyslexia. However, increasing neuroimaging evidence shows that reading disabilities result from the same impairments in phonological processing, regardless of FSIQ. In a previous study using functional magnetic resonance imaging (fMRI), both poor readers (PR; children with high FSIQ [dyslexia] and children with low FSIQ, all of whom were native English speakers) exhibited similar patterns of reduced activation in brain regions including the left parietotemporal and occipitotemporal regions during phonological processing [13]. In a different study, children who were all native English speakers with a discrepantly low reading ability relative to their FSIQ showed reduced activation in the left temporoparietal neocortex relative to the control children (typical readers [TR] without FSIQ discrepancy) [14]. In both studies, there was shared neurological atypicality in regions associated with phonological processing in children with poor reading ability that was substantially below the level expected for their FSIQ, regardless of FSIQ [13, 14].

This finding indicates that similar to dyslexia, academic achievement in children with poor reading ability might be lower than that in children with typical reading ability among children with borderline FSIQ. However, the relationship between academic achievement and reading ability in children with borderline FSIQ is unknown. Clarifying the relationship in children with borderline FSIQ between reading ability and academic achievement will benefit the field of special education in the future.

The purpose of the present study was to clarify the characteristics of academic achievement and reading ability in children with lower FSIQ (85 ≥ FSIQ ≥ 70) and poor reading ability, and differentiate these characteristics from those of children with higher FSIQ (FSIQ > 85) and poor reading ability. Unfortunately, up to this point, it has been impossible to evaluate their reading ability correctly because the poor reading ability is also associated with lower FSIQ [15]. The reading ability of children has typically been evaluated using reading data from TDC, contributing to the risks of overdiagnosis of children with lower FSIQ and poor reading ability, and underdiagnosis of gifted children with poor reading ability. Therefore, in this study, we first determined linear criteria on reading ability to separate PR from TR (see below, sections “Statistical analysis” “The classification of PR and TR considering FSIQ”). Furthermore, this study functioned to reveal the differences in clinical features of academic achievement and reading ability between children with lower FSIQ and poor reading ability and children with higher FSIQ and poor reading ability.

## Materials and methods

### Participants

In this prospective study, we enrolled 126 schoolchildren. All the children were referred to at the National Center Hospital, National Center of Neurology and Psychiatry (NCNP), Japan, between 2017 and 2020, based on teachers’ perceptions of poor written exam results and poor reading out aloud of textbooks compared to other children at school. All children were first-visit patients and had been referred by pediatricians in other hospitals or outpatient clinics, or by teachers in their schools or educational institutes. The age range of children in this study was 6–15 years (from elementary to junior high school). All the children lived and attended public schools in Japan, and had parents who spoke Japanese as their first language.

The children completed the Wechsler Intelligence Scale for Children, Fourth Edition (WISC-IV), based on which their intellectual level was assessed. Reading and writing achievement was determined using their reading and writing scores on the Kaufman Assessment Battery for Children, Second Edition (KABC-II; standard score = 10) [16]. In KABC-II reading and writing scores, children performed the task of reading and writing questions, which involve a mixture of Chinese characters and *hiragana* (Japanese syllabary). Chinese characters in the questions are arranged in the order children learn at school. The KABC-II is an important tool for diagnoses of children with dyslexia or academic proficiency evaluation because of the order of the questions that take into consideration the progress of grades [9]. We assessed whole writing and reading achievement using the KABC-II in children with poor reading ability, which was brought by poor reading ability.

The caregivers of the children completed the following questionnaires: a Japanese version of the Swanson, Nolan, and Pelham Rating Scale-IV (SNAP-IV) to assess symptoms of attention deficit hyperactivity disorder (ADHD) and oppositional defiant disorder (ODD) [17], and the Parent-Interview Autism Spectrum Disorder Rating Scale-Text Revision (PARS-TR) to assess symptoms of autism spectrum disorder (ASD) [18, 19].

The exclusion criteria were: (1) children with mild to profound intellectual disabilities (FSIQ < 70), and (2) children taking drugs or other treatments for attention deficit hyperactivity disorder (ADHD).

### Hiragana (Japanese syllabary) reading task procedure

The framework of the *hiragana* reading task was similar to that in Takeuchi et al.’s study [9, 20]. Each child was asked to complete four reading tasks that were written in *hiragana* script: a monomoraic syllable reading task, four-syllable word reading task, four-syllable non-word reading task, and short sentence reading task. This is the standardized reading examination used to diagnose developmental dyslexia in Japan [9]. The average reading time of each task is calculated from the data of TDC, and expansion of the reading time of more than 2SD is judged as an abnormal result [9]. Children with intelligence typical of their age who complete two or more *hiragana* reading tasks with a reading time greater than 2 standard deviations (SDs) above the average are diagnosed with dyslexia [9]. *Hiragana* is a Japanese syllabic script that is characterized by highly transparent sound-character correspondence, which can easily be processed in a phonological manner if linguistic processing is not restricted.

### Statistical analysis

Statistical analysis was conducted using JMP software (version 9.0.3; SAS Institute Inc., Cary, NC, USA). Children in the study were categorized as having either lower or higher FSIQ based on the threshold of an FSIQ score of 85, which was at the level of − 1 SD. Children were also categorized into PR or TR. First, we confirmed the relationship between FSIQ and reading time using simple regression analysis since a previous study demonstrated a linear relationship between FSIQ and reading ability [15]. Second, the reference value of reading time was calculated from the prediction formula, and children who exceeded the reference value in two or more reading tasks were classified as PR based on the diagnosis of dyslexia [9]. Finally, they were categorized into four groups based on FSIQ threshold and reading ability as follows: higher FSIQ and typical reading ability (H-TR), higher FSIQ and poor reading ability (H-PR), lower FSIQ and typical reading ability (L-TR), and lower FSIQ and poor reading ability (L-PR) groups.

We investigated how well the classification of TR and PR in the higher FSIQ group was obtained based on the SD value, which is a standard method for evaluating reading ability, and the reference value from the IQ adjusted- prediction formula, since children with a higher FSIQ were assumed to have normal intelligence.

Pearson’s χ^2^ test was performed to evaluate the proportional differences between 4 groups for gender and right-handedness. Pearson’s χ^2^ test was also performed to evaluate the proportional differences between higher FSIQ and lower FSIQ groups for number of children with PR. One-way analysis of variance (ANOVA) was performed to examine group differences in age at examination, three SNAP-IV items (inattention, hyperactivity, and ODD), and PARS-TR scores. Subsequently, post hoc analysis with Tukey’s honestly significant difference (HSD) test was performed.

Associations between reading and writing achievement (reading score and writing score from KABC-II) and age, intelligence, and *hiragana* reading tasks were assessed using Pearson’s product-moment correlation coefficients for the higher and lower FSIQ groups. In addition, associations between word reading and non-word reading tasks from *hiragana* reading tasks, and reading and writing achievement scores (reading score and writing score from KABC-II), age, and intelligence were also assessed using Pearson’s product-moment correlation coefficients for the higher and lower FSIQ groups. Statistical significance was set at *p* < 0.05.

## Results

### The classification of PR and TR considering FSIQ

Table 1 shows the clinical demographic parameters of the children enrolled in the study (i.e., 126 children identified as having poor academic performance in school).

**Table 1:** Clinical demographics of children

N	H-TR	H-PR	L-TR	L-PR	*p*-value
	50	41	18	17	-
Age (M, SD)	10.8 (2.5)	10.3 (2.1)	11.5 (2.5)	10.0 (2.7)	0.209
Male (n, %)	39 (78.0)	33 (80.5)	17 (94.4)	13 (76.5)	0.451
Right handiness (n, %)	44 (88.0)	39 (95.1)	17 (94.4)	15 (88.2)	0.602
SNAP-IV; Inattention (M, SD)	16.4 (16.0)	15.2 (6.7)	15.2 (6.8)	11.1 (7.0)	0.441
SNAP-IV; Hyperactivity (M, SD)	6.0 (5.4)	6.0 (5.2)	4.5 (3.5)	4.2 (5.8)	0.468
SNAP-IV; ODD (M, SD)	5.8 (5.3)	6.0 (6.4)	7.0 (8.1)	5.2 (6.6)	0.847
PARS-TR (M, SD)	5.1 (5.2)	4.9 (5.6)	5.5 (4.1)	6.1 (4.5)	0.871

*N* number, *M* mean, *SD* standard deviation, *H* high intellectual quotient, *L* low intellectual quotient, *TR* typical reader, *PR* poor reader, *SNAP-IV* Swanson, Nolan, and Pelham Rating Scale-IV, *ODD* oppositional defiant disorder, *PARS-TR* Parent-Interview Autism Spectrum Disorder Rating Scale-Text Revision

The simple linear regression analysis revealed a linear relationship between the FSIQ and SD value of reading time for each of the four reading tasks (monomoraic syllable reading task: *R*^2^ = 0.052, F = 6.818, *p* = 0.010, and reference value = 7.421–0.054×FSIQ; four-syllable word reading task: *R*^2^ = 0.068, F = 9.024, *p* = 0.003, and reference value = 9.276–0.073×FSIQ; four-syllable non-word reading task: *R*^2^ = 0.067, F = 8.888, *p* = 0.004, and reference value = 9.387–0.068×FSIQ; short sentence reading task: *R*^2^ = 0.040, F = 5.176, *p* = 0.025, and reference value = 8.085–0.061×FSIQ). The reference values for each FSIQ of every reading task were summarized in additional file 1. Children were divided into PR and TR based on the calculated reference value in their reading time relative to their FSIQ. There were 50, 41, 18, and 17 children in H-TR, H-PR, L-TR, and L-PR groups, respectively.

There was no difference in the proportion of PR between the higher FSIQ and lower FSIQ groups (45.1 % vs. 48.6 %) (*p* = 0.723). The classification using 2 SDs beyond the average score on reading tasks identified 38 poor readers in the higher FSIQ group, whereas the classification using the prediction formula identified 41 poor readers. Eighty-two out of 91 children (90.1 %) in the higher FSIQ group were identified in the same group from the two types of methods

### Children’s background

Table 1 shows the participant’s demographics. There was no difference based on sex, age at examination, or proportion of right-handedness among the four groups divided by IQ and reading ability. There was also no difference in the scores on the three items of SNAP-IV and PARS-TR scores among the four groups.

An additional table shows results of intelligence, reading ability, and reading and writing achievement of PR and TR in both higher and lower FSIQ groups (see Additional file 2).

### Factors related to reading and writing achievement and reading ability

Table 2 shows the associations between reading and writing achievement and participant demographics and between reading ability and participant demographics. In the higher FSIQ group, there were stronger positive relationships between reading scores from KABC-II and FSIQ, VCI, and WMI, and a stronger negative relationship between the reading score and each *hiragana* reading task. Furthermore, there were positive relationships between the writing scores from KABC-II and FSIQ and WMI, and stronger negative relationships between the writing score and monomoraic syllables, four-syllable word reading, and four-syllable non-word reading tasks. In contrast, in the lower FSIQ group, there was a negative relationship between reading and writing scores from KABC-II and age. In addition, there was a strong negative relationship between four-syllable non-word reading time and FSIQ, and VCI in the higher FSIQ group. The lower FSIQ group displayed a strong negative relationship between four-syllable word reading time and age.

**Table 2:** Associations between reading and writing achievement, reading ability, and intellectual level

	Higher FSIQ		Lower FSIQ	
	^b^Reading score	^b^Writing score	^b^Reading score	^b^Writing score
Age	-0.062	0.0487	**-0.5489**	**-0.3495**
WISC-IV: FSIQ	**0.4111**	**0.2738**	0.1119	-0.0403
WISC-IV: VCI	**0.409**	0.1648	0.2592	0.0204
WISC-IV: PRI	0.1948	0.1654	-0.1005	-0.1529
WISC-IV: WMI	**0.3486**	**0.2891**	0.3014	-0.062
WISC-IV: PRI	0.0385	0.0966	-0.3057	0.1872
Monomoraic syllables reading time	**-0.3678**	**-0.2421**	0.0377	-0.0505
Words reading time	**-0.4608**	**-0.342**	-0.122	-0.1127
Non-word reading time	**-0.482**	**-0.3133**	0.1963	-0.0585
Single sentences reading time	**-0.2649**	-0.1806	0.0891	-0.02
	Higher FSIQ		Lower FSIQ	
	^a^Four-syllable word	^a^Four-syllable non-word	^a^Four-syllable word	^a^Four-syllable non-word
Age	-0.1152	-0.0965	**-0.4493**	-0.3183
WISC-IV: FSIQ	-0.1951	**-0.2504**	0.102	0.0071
WISC-IV: VCI	-0.1399	**-0.2331**	-0.0226	-0.0615
WISC-IV: PRI	-0.1014	-0.0409	0.1585	0.1649
WISC-IV: WMI	-0.1893	-0.1688	-0.068	-0.0411
WISC-IV: PRI	-0.0478	-0.174	0.1472	-0.1063

All values are Pearson's correlation coefficients (*r*)

*p* < 0.05 is in bold

*WISC-IV* Wechsler Intelligence Scale for Children, Fourth Edition, *FSIQ* full-scale intellectual quotient, *VCI* verbal comprehension index, *PRI* perceptual reasoning index, *WMI* working memory index, *PSI* processing speed index

^a^Reading times for each reading task

^b^Reading score and writing score from KABC-II

## Discussion

To the best of our knowledge, this is the first study to clarify the clinical characteristics of Japanese children with lower FSIQ (85 ≥ FSIQ ≥ 70) and poor reading ability.

There was no difference in the proportion of PR between higher and lower FSIQ groups. Poor reading and writing achievement from KABC-II in the lower FSIQ group was associated with older age, but not with reading ability or FSIQ. In contrast, poor reading and writing achievement in the higher FSIQ group was associated with low FSIQ and low scores on its subscales, and poor reading ability.

### The classification of PR and TR considering FSIQ

In our study, participants were evaluated depending on FSIQ. Similar to previous studies, reading difficulty was evaluated by reading comprehension (IQ) and phonological awareness [10, 21]. Children were diagnosed with dyslexia if their reading ability was much lower than their other cognitive abilities despite having intelligence typical of children their age. In addition, among children with low reading comprehension, some children also had low phonological awareness [10, 11, 21]. In a previous fMRI study, regardless of FSIQ, PR had similar kinds of reading disabilities concerning phonological processing (reduced activation in the left parietotemporal and occipitotemporal regions) [13]. Namely, in PR, the relationship between FSIQ and reading ability, which was lower than that expected based on the obtained FSIQ, was relative. Furthermore, since a previous study revealed that FSIQ has a positive relationship with decoding and visual processing speed, which are significant factors of reading ability, a linear relationship between FSIQ and reading time was presumed [15]. Therefore, it is necessary to set the reading ability threshold according to FSIQ, and our method for classifying children as PR and TR considering FSIQ was valuable, especially for those borderline FSIQ and gifted children, whose evaluation of reading ability by SD value was easily distorted.

Since Japanese children are diagnosed with dyslexia based on reading time > 2 SDs on two or more out of the four *hiragana* tasks, children were also diagnosed based on the number of tasks above the reference value adjusted by FSIQ in this study. In fact, the reading times of the four tasks were first converted to SD value for the diagnosis of dyslexia. Furthermore, because there is a linear correlation between the FSIQ and SD values, the FSIQ-adjusted reference values were calculated from the raw SD values of *hiragana* reading tasks. In addition, since diagnoses by 2 SDs above the average value and diagnoses by reference values were the same in 90 % of children with higher FSIQ, the clinical practicality of our new diagnostic method has been confirmed.

In this study, we classified children identified as having low academic performance in school into PR and TR considering FSIQ. The diagnostic tools used for dyslexia (*hiragana* reading tasks) also helped find out children with lower FSIQ and poor reading ability.

### The relationship between reading ability and reading and writing achievement

In our study, the poor reading ability was associated with poor reading and writing achievement in the higher FSIQ group. A previous study demonstrated that the achievement gap caused by poor reading ability between TDC and children with dyslexia appeared as early as the first grade, and persisted not only in reading ability, but also in the verbal components of IQ (i.e., vocabulary, information, comprehension, and similarities subtests; WISC-Revised) [4]. Low levels of literacy achievement at the end of compulsory school education in Finland also significantly increased the likelihood of delayed graduation from upper secondary schools [5]. In particular, even in a study of university students with dyslexia, one-fifth of students had very low academic achievement [6]. As in previous studies, the present study revealed that poor reading ability leads to poor reading and writing achievement in children with higher FSIQ, who were identified as having poor academic performance in school. Importantly, this relationship was revealed only in the higher FSIQ group.

### The relationship between age and reading and writing achievement, and between age and reading ability

In the lower FSIQ group, lower reading and writing achievement was associated with older age at examination, but not with reading ability.

As mentioned in section “The relationship between reading ability and reading and writing achievement”, previous studies have shown that poor reading ability in children with dyslexia results in poorer academic achievement than TDC from the first grade onwards [4–6]. Our study’s results in the higher FSIQ group are consistent with this finding. Furthermore, in our study, reading and writing achievement scores for those in the lower FSIQ group were lower regardless of reading ability than scores for TR in the higher FSIQ group. In addition, there was no relationship between reading ability and reading and writing achievement in the lower FSIQ group. This finding reveals that poor reading ability in the lower FSIQ group did not cause poor reading and writing achievement, unlike in the higher FSIQ group. The difference in reading and writing achievement between the lower and higher FSIQ groups might have resulted from the stronger deterioration in reading and writing achievement with age in the lower FSIQ than in the higher FSIQ group.

Concerning the relationship between phonological deficits and age, the persistence of phonological deficits in children and adults with dyslexia has been demonstrated in previous longitudinal studies [4, 22, 23]. Furthermore, considering the difference between TDC, children with dyslexia, and children with low FSIQ and poor reading ability in the relationship between chronological age and reading ability, TDC showed linear development, children with dyslexia showed atypical and non-linear development, and children with low IQ and poor reading ability showed linear development (with a developmental delay compared to TDC) [11]. Therefore, the negative relationship between reading ability and age in the PR in the lower FSIQ group might have resulted from developmental delays of the PRs’ phonological deficits.

According to the previous questionnaire studies in Japan [8, 24], the percentage of children with a fundamental reading difficulty in public elementary school classes decreased as they progressed to higher grades, whereas, the percentage of children whose overall Japanese and mathematics learning proficiency was delayed by more than two grades increased as they progressed to higher grades. It was shown that the proportion of children who deviated from children with average academic achievement increased with age, although age-related improvement in reading ability was observed. In other words, the relationship between age and academic achievement was unknown in previous studies, certain people have been identified whose academic performance deteriorates with age.

A previous study revealed that the persistent academic achievement gap had serious consequences for readers with dyslexia, including lower rates of high school graduation, higher levels of unemployment, and lower earnings because of lowered college attainment [4]. In our study, low reading and writing achievement in the lower FSIQ group was found out not only in PR but also in TR (Additional file 2); moreover, age-related deterioration was observed. Therefore, these negative results might lead to more serious outcomes for the education or employment of children in the lower FSIQ group than for PR in the higher FSIQ group.

Unfortunately, background factors of age-related deterioration in academic achievement and reading ability were not clear in this study. However, one of the reasons might be the difficulty in accumulating learning because children with both higher IQ and lower IQ attend the same classes in Japanese public schools, such that children with lower FSIQ have fewer opportunities to receive appropriate educational support than children with higher FSIQ.

### Special education for children with lower FSIQ

Concerning the special education of children with lower FSIQ, earlier special support, and independent evaluations of reading ability and reading and writing achievement by teachers may be important because of the lack of a relationship between reading ability and reading and writing achievement in children with lower FSIQ. Furthermore, previous studies revealed that early and evidence-based reading instruction and teacher training offer the potential to reduce and perhaps even close the achievement gap between children with dyslexia and TR and bring their trajectories closer over time [4, 25, 26]. Since PR in the lower FSIQ group were more likely to demonstrate lower reading achievement at an older age, these children need not only special support similar to that offered to PR in the higher FSIQ group but also more careful educational interventions and more frequent evaluations of their reading and writing achievement than PR in the higher FSIQ group.

## Limitations

This study had several limitations. First, the number of children analyzed was small, affecting the generalizability of the results. Second, the identification of whether children had poor academic performance in school was based on the subjective judgment of teachers and referral doctors, rather than an objective index. Third, our findings might be specific to Japanese-speaking contexts, and therefore, ungeneralizable to children speaking other languages. Fourth, observation of children’s own adaptive behavior to their learning and educational support could not be considered insufficient. However, there was no difference in the severity of ADHD and ASD symptoms using SNAP-IV and PARS-TR between H-TR, H-PR, L-TR, and L-PR groups in our study. Therefore, there might not be a decrease in adaptive behavior brought by the trait of NDDs, although it was well known that severely autistic children found it more difficult to adapt to overall daily life than TDC in previous studies [27, 28].

## Conclusions

In our study, poor reading and writing achievement in the higher FSIQ group was associated with low FSIQ and low scores on its subscales as well as poor reading ability, as also shown in previous studies. However, poor reading and writing achievement was associated with older age, but not with poor reading ability in the lower FSIQ group. Therefore, children with lower FSIQ need appropriate educational interventions based on independent assessments of their reading and writing achievement and reading ability. Moreover, they need more frequent evaluations of their reading and writing achievement than children with higher FSIQ and poor reading ability since they are more likely to demonstrate lower reading achievement at an older age.

## Supplementary Information

**Additional file 1.** The results of the reference value from the IQ adjusted-prediction formula.

**Additional file 2.** Examination results for intelligence, reading ability, and reading and writing achievement

## Data Availability

The datasets generated and/or analyzed during the current study are not publicly available (study under progress) but are available from the corresponding author on reasonable request.

## Abbreviations

FSIQ: Full-scale intellectual quotient
TDC: Typically developing children
ANOVA: Analysis of variance
FMRI: Functional magnetic resonance imaging
NCNP: National Center of Neurology and Psychiatry
WISC-IV: Wechsler Intelligence Scale for Children, Fourth Edition
KABC-II: Kaufman Assessment Battery for Children, Second Edition
SNAP-IV: Swanson, Nolan, and Pelham Rating Scale-IV
ODD: Oppositional defiant disorder
PARS-TR: Parent-Interview Autism Spectrum Disorder Rating Scale-Text Revision
ASD: Autism spectrum disorder
ADHD: Attention deficit hyperactivity disorder
SD: Standard deviation
TR: Typical readers
PR: Poor readers
HSD: Honestly significant difference
