# Supplementary material for: Clinical profile of reading ability and reading and writing achievement of children with borderline full-scale intellectual quotient: a prospective study
Source: BMC Pediatr. 2021 Sep 7;21:389. doi: 10.1186/s12887-021-02865-z (PMC8422711; doi:10.1186/s12887-021-02865-z)
Supplement: Supplementary file 1 — Additional file 1. The results of the reference value from the IQ adjusted-prediction formula. [file 12887_2021_2865_MOESM1_ESM.docx]

Additional file 1. The results of the reference value from IQ adjusted-prediction formula

| FSIQ | Monomoraic syllable | Four-syllable word | Four-syllable non-word | Short sentence |
| --- | --- | --- | --- | --- |
| 70 | 3.61 | 4.18 | 4.05 | 3.83 |
| 71 | 3.56 | 4.11 | 3.98 | 3.77 |
| 72 | 3.50 | 4.03 | 3.92 | 3.71 |
| 73 | 3.45 | 3.96 | 3.85 | 3.65 |
| 74 | 3.39 | 3.89 | 3.78 | 3.59 |
| 75 | 3.34 | 3.82 | 3.71 | 3.52 |
| 76 | 3.29 | 3.74 | 3.64 | 3.46 |
| 77 | 3.23 | 3.67 | 3.57 | 3.40 |
| 78 | 3.18 | 3.60 | 3.51 | 3.34 |
| 79 | 3.12 | 3.52 | 3.44 | 3.28 |
| 80 | 3.07 | 3.45 | 3.37 | 3.22 |
| 81 | 3.01 | 3.38 | 3.30 | 3.16 |
| 82 | 2.96 | 3.31 | 3.23 | 3.10 |
| 83 | 2.91 | 3.23 | 3.16 | 3.04 |
| 84 | 2.85 | 3.16 | 3.10 | 2.98 |
| 85 | 2.80 | 3.09 | 3.03 | 2.92 |
| 86 | 2.74 | 3.02 | 2.96 | 2.86 |
| 87 | 2.69 | 2.94 | 2.89 | 2.80 |
| 88 | 2.63 | 2.87 | 2.82 | 2.73 |
| 89 | 2.58 | 2.80 | 2.75 | 2.67 |
| 90 | 2.52 | 2.72 | 2.69 | 2.61 |
| 91 | 2.47 | 2.65 | 2.62 | 2.55 |
| 92 | 2.42 | 2.58 | 2.55 | 2.49 |
| 93 | 2.36 | 2.51 | 2.48 | 2.43 |
| 94 | 2.31 | 2.43 | 2.41 | 2.37 |
| 95 | 2.25 | 2.36 | 2.34 | 2.31 |
| 96 | 2.20 | 2.29 | 2.28 | 2.25 |
| 97 | 2.14 | 2.21 | 2.21 | 2.19 |
| 98 | 2.09 | 2.14 | 2.14 | 2.13 |
| 99 | 2.03 | 2.07 | 2.07 | 2.07 |
| 100 | 1.98 | 2.00 | 2.00 | 2.00 |
| 101 | 1.93 | 1.92 | 1.93 | 1.94 |
| 102 | 1.87 | 1.85 | 1.87 | 1.88 |
| 103 | 1.82 | 1.78 | 1.80 | 1.82 |
| 104 | 1.76 | 1.70 | 1.73 | 1.76 |
| 105 | 1.71 | 1.63 | 1.66 | 1.70 |
| 106 | 1.65 | 1.56 | 1.59 | 1.64 |
| 107 | 1.60 | 1.49 | 1.53 | 1.58 |
| 108 | 1.55 | 1.41 | 1.46 | 1.52 |
| 109 | 1.49 | 1.34 | 1.39 | 1.46 |
| 110 | 1.44 | 1.27 | 1.32 | 1.40 |
| 111 | 1.38 | 1.20 | 1.25 | 1.34 |
| 112 | 1.33 | 1.12 | 1.18 | 1.28 |
| 113 | 1.27 | 1.05 | 1.12 | 1.21 |
| 114 | 1.22 | 0.98 | 1.05 | 1.15 |
| 115 | 1.16 | 0.90 | 0.98 | 1.09 |
| 116 | 1.11 | 0.83 | 0.91 | 1.03 |
| 117 | 1.06 | 0.76 | 0.84 | 0.97 |
| 118 | 1.00 | 0.69 | 0.77 | 0.91 |
| 119 | 0.95 | 0.61 | 0.71 | 0.85 |
| 120 | 0.89 | 0.54 | 0.64 | 0.79 |
| 121 | 0.84 | 0.47 | 0.57 | 0.73 |
| 122 | 0.78 | 0.39 | 0.50 | 0.67 |
| 123 | 0.73 | 0.32 | 0.43 | 0.61 |
| 124 | 0.67 | 0.25 | 0.36 | 0.55 |
| 125 | 0.62 | 0.18 | 0.30 | 0.48 |
| 126 | 0.57 | 0.10 | 0.23 | 0.42 |
| 127 | 0.51 | 0.03 | 0.16 | 0.36 |
| 128 | 0.46 | -0.04 | 0.09 | 0.30 |
| 129 | 0.40 | -0.12 | 0.02 | 0.24 |
| 130 | 0.35 | -0.19 | -0.05 | 0.18 |
