# Supplementary material for: Clinical profile of reading ability and reading and writing achievement of children with borderline full-scale intellectual quotient: a prospective study
Source: BMC Pediatr. 2021 Sep 7;21:389. doi: 10.1186/s12887-021-02865-z (PMC8422711; doi:10.1186/s12887-021-02865-z)
Supplement: Supplementary file 2 — Additional file 2. Examination results for intelligence, reading ability, and reading and writing achievement [file 12887_2021_2865_MOESM2_ESM.docx]

Additional file 2. Examination results for intelligence, reading ability, and reading and writing achievement

|  | Higher FSIQ | | Lower FSIQ | |
| --- | --- | --- | --- | --- |
|  | TR | PR | TR | PR |
| WISC-Ⅳ; FSIQ (M, SD) | 100.5 (11.0) | 96.9 (8.4) | 77.4 (7.2) | 78.2 (4.9) |
| WISC-Ⅳ; VCI (M, SD) | 104.1 (13.4) | 100.9 (10.6) | 84.4 (9.4) | 85.0 (11.6) |
| WISC-Ⅳ; PRI (M, SD) | 103.5 (13.2) | 101.5 (12.7) | 81.9 (11.9) | 83.8 (10.7) |
| WISC-Ⅳ; WMI (M, SD) | 94.5 (17.5) | 89.6 (10.6) | 77.6 (9.5) | 76.6 (10.1) |
| WISC-Ⅳ; PSI (M, SD) | 93.0 (11.2) | 93.7 (10.5) | 81.5 (8.0) | 82.4 (10.0) |
| Monomoraic syllables SD^†^ (M, SD) | 0.4 (0.9) | 3.9 (3.6) | 1.3 (1.1) | 5.5 (3.4) |
| Four-syllable words SD^†^ (M, SD) | 0.6 (1.1) | 5.6 (4.0) | 2.4 (1.7) | 6.8 (3.3) |
| Four-syllable non-words SD^†^ (M, SD) | 1.1 (1.7) | 4.2 (3.2) | 1.4 (1.0) | 6.5 (5.6) |
| Short sentences SD^†^ (M, SD) | 0.3 (1.4) | 4.0 (5.0) | 1.1 (1.6) | 6.1 (4.9) |
| KABC-Ⅱ; Reading (M; SD) | 9.7 (3.1) | 6.8 (3.0) | 6.8 (2.7) | 6.2 (2.6) |
| KABC-Ⅱ; Writing (M; SD) | 7.6 (2.8) | 5.9 (3.0) | 5.7 (2.3) | 5.3 (1.7) |

^†^Reading times for each reading task

FSIQ: full scale intellectual quotient; TR: typical reader; PR: poor reader; WISC-IV: Wechsler Intelligence Scale for Children, Fourth Edition; VCI: verbal comprehension index; PRI: perceptual reasoning index; WMI: working memory index; PSI: processing speed index; KABC-II: Kaufman Assessment Battery for Children, Second Edition; M: mean; SD: standard deviation
